# Life cycle environmental impact assessment for battery-powered electric vehicles at the global and regional levels

**DOI:** 10.1038/s41598-023-35150-3

**Published:** 2023-05-16

**Authors:** Hongliang Zhang, Bingya Xue, Songnian Li, Yajuan Yu, Xi Li, Zeyu Chang, Haohui Wu, Yuchen Hu, Kai Huang, Lei Liu, Lai Chen, Yuefeng Su

**Affiliations:** 1 School of Management and Economics, Center for Energy and Environmental Policy Research, Beijing Institute of Technology, Beijing, 100081 China; 2 Department of Energy and Environmental Materials, School of Materials Science and Engineering, Beijing Institute of Technology, Beijing, 100081 China; 3 Beijing Institute of Technology Chongqing Innovation Center, Chongqing, 401120 China; 4 Beijing Automotive Technology Center, Beijing, 100163 China; 5 College of Environmental Science and Engineering, Beijing Forestry University, Beijing, 100083 China; 6 Department of Civil and Resource Engineering, Dalhousie University, Halifax, B3H4R2 Canada

**Keywords:** Environmental impact, Batteries

## Abstract

As an important part of electric vehicles, lithium-ion battery packs will have a certain environmental impact in the use stage. To analyze the comprehensive environmental impact, 11 lithium-ion battery packs composed of different materials were selected as the research object. By introducing the life cycle assessment method and entropy weight method to quantify environmental load, a multilevel index evaluation system was established based on environmental battery characteristics. The results show that the Li–S battery is the cleanest battery in the use stage. In addition, in terms of power structure, when battery packs are used in China, the carbon footprint, ecological footprint, acidification potential, eutrophication potential, human toxicity cancer and human toxicity noncancer are much higher than those in the other four regions. Although the current power structure in China is not conducive to the sustainable development of electric vehicles, the optimization of the power structure is expected to make electric vehicles achieve clean driving in China.

## Introduction

The transportation industry is developing rapidly and plays a particularly important role in economic and social development^1^. At the same time, it also consumes many fossil fuels and causes serious environmental pollution^2^. IEA (2019) reports that approximately one-third of global CO_2_ emissions are caused by the transport sector^3, 4^. As the world's largest carbon dioxide emitter, China has had to deal with serious energy and environmental problems in recent years^5^. To alleviate the huge energy demand and environmental pressure in the global transportation industry, the electrification of the transportation sector is considered one of the key measures to reduce the emission of pollutants^6–8^. Therefore, the development of clean and sustainable energy vehicles, especially electric vehicles (EVs), has become a promising choice in the automotive industry^9^.

In this context, in September 2001, new energy vehicles were included in the national "863" plan, after which the "major science and technology project of EVs" was launched, marking the start of China's EV research and development. Since the 12th Five-Year Plan (2010–2015), the Chinese government has decided to promote the use of EVs to make travel cleaner. However, the severe economic crisis has left all countries facing the problems of an energy crisis, rising fossil fuel prices, high unemployment and rising inflation, which affect the mentality of society, people's spending power and government decision-making. Therefore, people's recognition and acceptance of EVs is not high, which hinders the early market diffusion of EVs^10^. This comes at a time when the Chinese government has rolled out a series of policy measures and financial incentives to promote the development of EVs^11^. Since 2013, China has implemented a policy of subsidizing EV purchases^12^. From January 1, 2021, to December 31, 2022, new energy vehicles purchased will be exempted from the vehicle purchase tax. In the short term, government measures such as subsidies to narrow the price gap and the development of charging infrastructure will boost EV' consumption^13^.

**Figure 1:**
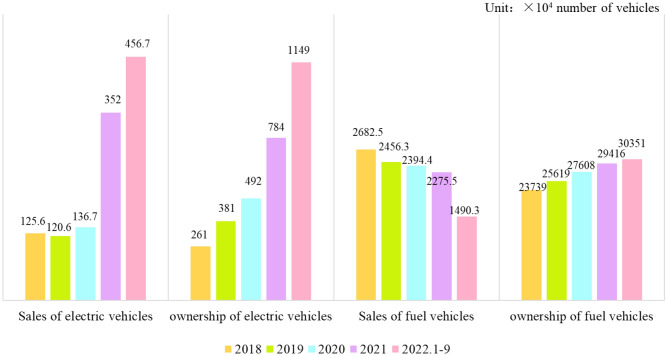
Sales and ownership of EVs and fuel vehicles from 2018 to September 2022.

In recent years, the sales and ownership of EVs and fuel vehicles in China are have been shown in Fig. 1. It can be seen that the sales and ownership of EVs in China are on the rise, especially in the past two years. In contrast, the fuel car sales continue to decline, and the growth trend of ownership slows down. In other words, with the guidance of regulations and the awakening of environmental awareness, the shift in sales of conventional fuel cars is the opposite of that of EVs, and the popularity rate of EVs is increasing rapidly^7, 14^. Currently, lithium-ion batteries (LIBs) are the first choice in the EV field due to their advantages of light weight, great performance, high energy density and high output power^15–19^. Additionally, LIBs, as the main technology in battery energy storage systems^20^, also have great potential for energy sustainability and significant reductions in carbon emissions^21^.

In the process of promotion, EVs are sometimes considered to be zero-emission vehicles, but their production and use of battery packs will have a great impact on the environment. Therefore, recent studies have focused more on the environmental benefits of EVs^22^. There is much research on the three stages of EVs: production, use and recycling. For example, Feng et al.^23^ took the three most widely used lithium nickel cobalt manganese oxide (NCM) batteries and lithium iron phosphate (LFP) batteries in the EV market in China as the research object, and conducted a specific analysis of the three stages of power battery production, use and recycling based on life cycle assessment (LCA). The result shows that LFP batteries have better environmental performance than NCM batteries under overall conditions, but the energy efficiency in the use phase is inferior to NCM batteries, which have greater recycling value.

For the production stage, Hao et al.^24^ estimated GHG emissions from the production of LIBs in China by establishing a LCA framework. For the three types of most commonly used LIBs: the LFP battery, the NMC battery and the LMO battery, the GHG emissions from the production of a 28 kWh battery are 3061 kg CO_2_-eq, 2912 kg CO_2_-eq and 2705 kg CO_2_-eq, respectively.

For the use phase, Zeng et al.^25^ used BYD Qin Pro series models in China as an example to compare the environmental impact of pure BEVs and plug-in hybrid EVs with traditional internal combustion engine vehicles. The result shows that compared to the gasoline ICEV, BEVs and plug-in hybrid EVs driven by the current average power structure in China reduce global warming potential by 23% and 17%, respectively.

For the recovery and reuse stage, Koroma et al.^26^ conducted a LCA for three different scenarios combined with battery recycling and found that recycling reduced the climate impact of EVs by almost 8%, with human toxicity and mineral resource scarcity reduced by approximately 22% and 25%, respectively. Yang et al.^27^ used LCA to study the environmental feasibility of reusing waste LIBs in communication base stations. The results show that in all selected categories, the secondary use of EV LIBs has less environmental impact than the use of lead-acid batteries.

EVs are being called "zero-emission" vehicles, but there is a new argument for that common belief. Pure EVs have no direct greenhouse gas emissions in the use process, but their environmental burden will be indirectly transferred to the power structure. That is, the power structure of charging is an important factor affecting each environmental index. In addition, there are almost no articles that have conducted a separate study on the use stage of EVs, so it is necessary to analyze their driving state on the road. Finally, more valuable advice is needed on how the electricity structure can be adjusted to get closer to "zero emissions" on the road. Therefore, the environmental impact of battery packs in the use stage is worth further study. From this point of view, this study focuses on the impact of battery use and establishes an LCA integrated environmental system.

In this work, based on footprint family, resource depletion and toxic damage indicators, 11 types of EV battery packs and five regions were selected to evaluate the environmental burden of different types of LIBs, and to measure the superiority of battery pack categories and the importance of power network structure. In addition, a dimensionless environmental characteristic index was established to assess the comprehensive environmental impact of the battery pack. The results showed that the Li–S battery is the cleanest battery in the use stage. In addition, the electrical structure of the operating area is an important factor for the potential environmental impact of the battery pack. In terms of power structure, coal power in China currently has significant carbon footprint, ecological footprint, acidification potential and eutrophication potential. Although the current power structure in China is not conducive to the sustainable development of electric vehicles, the optimization of the power structure is expected to make electric vehicles achieve clean driving in China.

## Methods

Due to the different power structures in different regions, the decarbonization capacity of the power sector is not consistent, and the environmental impacts are also discrepant. In the use stage, this work assumes that the EV travels in five different regions to analyze the influence of regional power structure on the environmental characteristics of the battery pack. Compared with other models, the mini-car has the characteristics of smaller battery capacity, less energy demand, miniaturization and convenience, which is suitable for short distance driving and conducive to promotion. Therefore, this study only explored the comprehensive environmental impact of mini EVs (the mini-car weighs 1100 kg, the battery capacity is 17.7 kWh, and the energy demand is 96.8 Wh km^−1^).

### LCA method

As a scientific method to evaluate the energy demand and the emissions associated with products’ life cycles^28^, LCA has been widely used in product environmental characteristic analysis and decision support. LCA is divided into four stages: objective and scope determination, inventory analysis, evaluation impact analysis and results, and interpretation or optimization of evaluation results^29^. In this study, the footprint family, resource depletion and toxic damage of EV battery packs were evaluated comprehensively by the LCA method.

In this study, 11 kinds of batteries were selected as research objects to analyze their environmental impact under the power structure in 5 regions. The scope of the study is the EV use process, which does not involve the production of the car and battery but only the process of charging the battery and running the car on the road. A certain distance was taken as the evaluation unit of the environmental impact of the battery. When the EV of different batteries travels the same mileage, their respective battery capacity is different. The power comes from the electric energy that the EV absorbs while charging. That power, in turn, comes from energy sources such as coal, nuclear or hydropower. Therefore, it can be seen that a certain driving distance, under the support of different batteries, can correspond to their respective power. Therefore, we define the functional unit as the distance traveled per unit.

Since the commonly used commercial battery pack types are LFP and NMC, two kinds of LFP (according to the different compositions and proportions of LFP cathode materials), three kinds of NMC (according to the different composition ratios of the three active materials of nickel, cobalt and manganese and the different proportions of cathode materials) and two kinds of NMC batteries combined with nanoanode materials (silicon nanowires and silicon nanotubes) were selected. In addition, one kind of battery pack with LMO as a positive active material, one kind of composite cathode material battery containing LMO and NMC and two kinds of LIBs containing sulfur were also selected. Therefore, the research objects of this study were eleven different types of LIB packs, including LFP_x_-C^30^, LFP_y_-C^31^, NMC-C^31^, NMC_442_-C^30^, NMC_111_-C^32^, NMC-SiNT^33^, NMC-SiNW^34^, LMO-C^35^, LMO/NMC-C^36^, Li-S^37^ and FeS_2_SS^38^.

Battery packs can be divided into four categories according to their components, namely LFP, NMC, LMO and LMB. Specific information:

LFP: LFP_x_-C, lithium iron phosphate oxide battery with graphite for anode, its battery pack energy density was 88 Wh kg^−1^ and charge‒discharge energy efficiency is 90%; LFP_y_-C, lithium iron phosphate oxide battery with graphite for anode, x and y only represent different battery types, its charge‒discharge efficiency is 95% and electricity consumption is 15 kWh per 100 km.

NMC: NMC-C, lithium-nickel manganese cobalt oxide (LiNi_x_Mn_y_Co _(1-x–y)_ O_2_) coupled with a graphite anode material, its charge‒discharge efficiency is 99% and electricity consumption was 13 kWh per 100 km; NMC_442_-C, lithium-nickel manganese cobalt oxide (LiNi_0.4_Mn_0.4_Co_0.2_O_2_) coupled with a graphite anode material, battery pack energy density is 112 Wh kg^−1^ and charge‒discharge energy efficiency is 90%; NMC_111_-C, lithium-nickel manganese cobalt oxide (LiNi_0.33_Mn_0.33_Co_0.33_O_2_) coupled with a graphite anode material, its energy capacity is 26.6 kWh and battery efficiency is 95% to 96%; NMC-SiNT, lithium-nickel manganese cobalt oxide (LiNi_x_Mn_y_Co _(1-x–y)_ O_2_) coupled with a silicon nanotube anode material, its gravimetric energy density is 199 Wh kg^−1^ and charge‒discharge efficiency is 90%; NMC-SiNW, lithium-nickel manganese cobalt oxide (LiNi_x_Mn_y_Co _(1-x–y)_ O_2_) coupled with a silicon nanowire anode material, the battery pack has a total weight of 120 kg and energy capacity of 43.2 kWh.

LMO: LMO-C, lithium manganese oxide (LiMn_2_O_4_) coupled with a graphite anode material, the battery weight is 300 kg and the battery capacity was 34.2 kWh; LMO/NMC-C, lithium manganese oxide coupled with a graphite anode material (LiMn_2_O_4_ and LiNi_0.4_Mn_0.4_Co_0.2_O_2_), the nominal capacity of which is 11.4 kWh and can to be used for approximately 140,000 km of driving;

LMB: Li–S, lithium metal coupled with elemental sulfur, its total energy capacity is 61.3 kWh and charging efficiency is 95%; FeS_2_SS, solid-state lithium battery with iron sulfide (FeS_2_) for cathode; lithium metal for the anode; and lithium sulfide (Li_2_S) and phosphorous pentasulfide (P_2_S_5_) for solid-state electrolyte, its specific capacity of 182 Wh kg^−1^ and energy capacity is 80 kWh.

Studies assessing the environmental impacts of LIBs assume total driving distances between 150,000 km and 200,000 km^34^. In this study, it is assumed that the EV’s battery has a serves range of 180,000 km, and no replacement of batteries is considered during the use period. The boundary range of the study is the use stage of the battery pack, so the functional unit is determined to be 1 km, that is, the environmental impact of the power battery pack in the use stage is calculated based on the unit running distance. The basic scenario parameters are listed in Table 1.

**Table 1:** The basic scenario parameters in the use stage.

Life cycle phase	Parameters	Base case
Use	Battery charging efficiency	95%
	Efficiency of the charger	96%^39^
	Weight-energy relationship	30%^40^
	Lifetime (km)	180,000^39^

### Power structure and operation calculation of the power battery pack in the use phase

In the operation phase, the regional analysis emphasizes the difference in the influence of different power combinations on the analysis results. Therefore, in the use of EV battery packs, the power supply structure will affect the environmental emissions to a large extent. The regions of the use stage of EV are determined in five regions for analysis, including Global, China, Japan, Europe and the US.

In the use stage, the power loss of the battery (to provide power for EV transportation), the extra power required by the vehicle to transport the battery, and the energy consumed during the vehicle operation were considered. The battery usage process is calculated based on the assumptions of the base scenario (Table 1).

Power loss (${EL}_{be}$) due to battery charging efficiency:

1 $${EL}_{be}={D}_{v}\times {CEL}_{drm}\times (1-\eta c)$$

where ${EL}_{be}$ represents the power loss caused by battery charging, kWh; ${D}_{v}$ is the mileage of the electric vehicle, km; and ${CEL}_{drm}$ represents EV's power consumption per kilometer, kWh km^−1^.$\eta c$ is the efficiency of a battery, %.

Extra power (*EL*_*ex*_) from the transportation of battery:

2 $${EL}_{ex}={W}_{b}/{W}_{v}\times {CEL}_{w}\times {CEL}_{drm}/\eta c\times {D}_{v}$$

where ${EL}_{ex}$ represents the extra power required to transport the battery, kWh; ${W}_{b}$ is the weight of the battery pack, kg; ${W}_{v}$ is the weight of the EV, kg; and ${CEL}_{w}$ represents the direct relationship between energy consumption and battery transport (weight-energy ratio: 30% in the base scenario), %.

The energy consumed (*ELu*)during battery life is:

3 $${EL}_{u}={CA}_{b}\times INT({D}_{v}/{D}_{r})$$

where* Elu represents* the energy consumed during battery life, kWh; ${CA}_{b}$ is battery pack capacity, kWh; and ${D}_{r}$ stands for the mileage of EV in a cycle, km charge^−1^.

**Figure 2:**
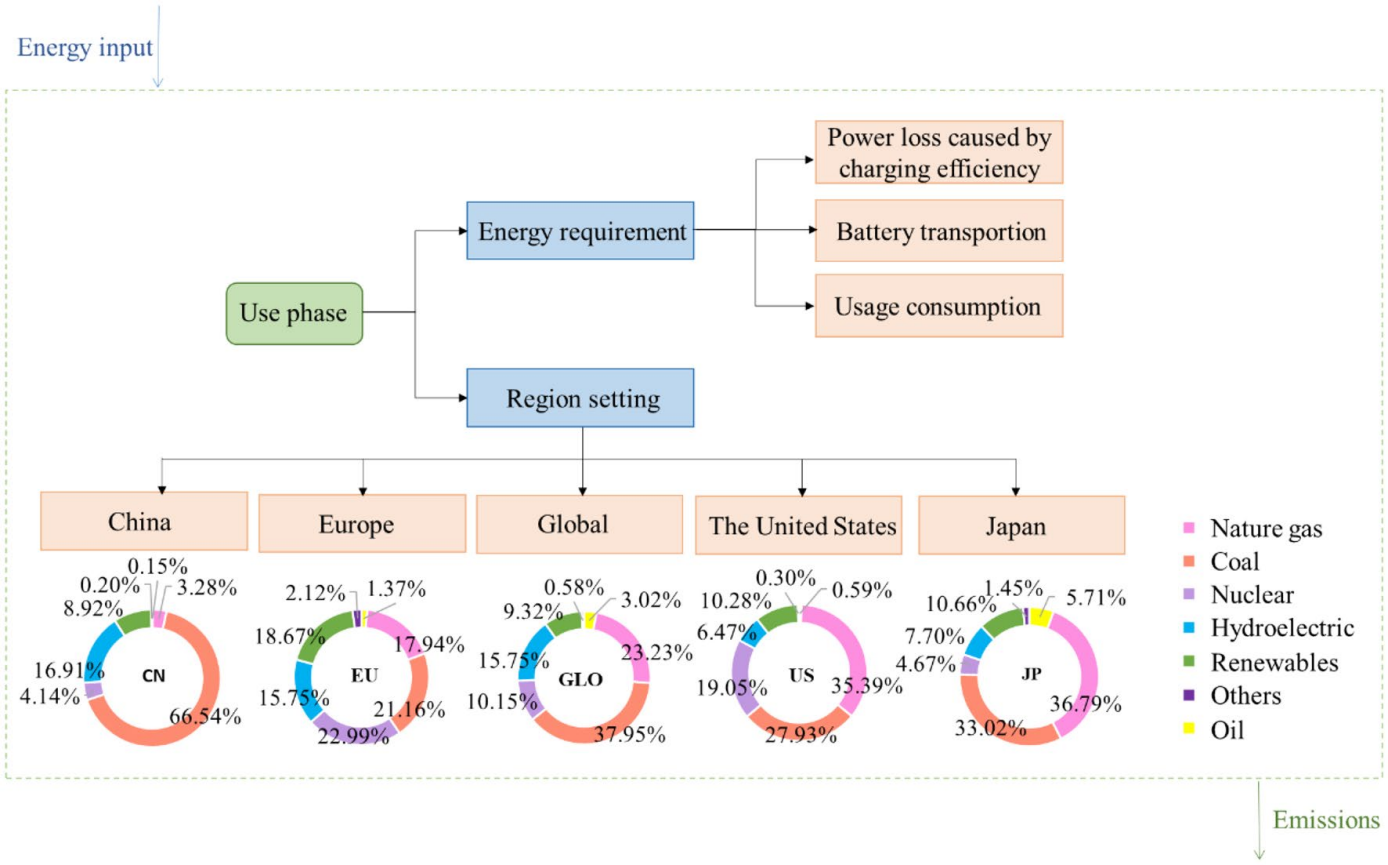
The frame diagram of the use phase and electricity generation structure in different regions in 2018 (Data source: http://bp.com/statsreview).

The energy in the use stage of the battery pack consists of power loss, extra power and power consumption. The frame diagram of the use phase and electricity generation structure in different regions in 2018 are shown in Fig. 2.

According to the above formula, the total electric energy consumed by electric vehicles in the driving stage is calculated, and then fed into Simapro software. According to the power structure of different regions, the three-level index value of emissions in the power generation process can be calculated.

### Comprehensive environmental assessment indicators

**Figure 3:**
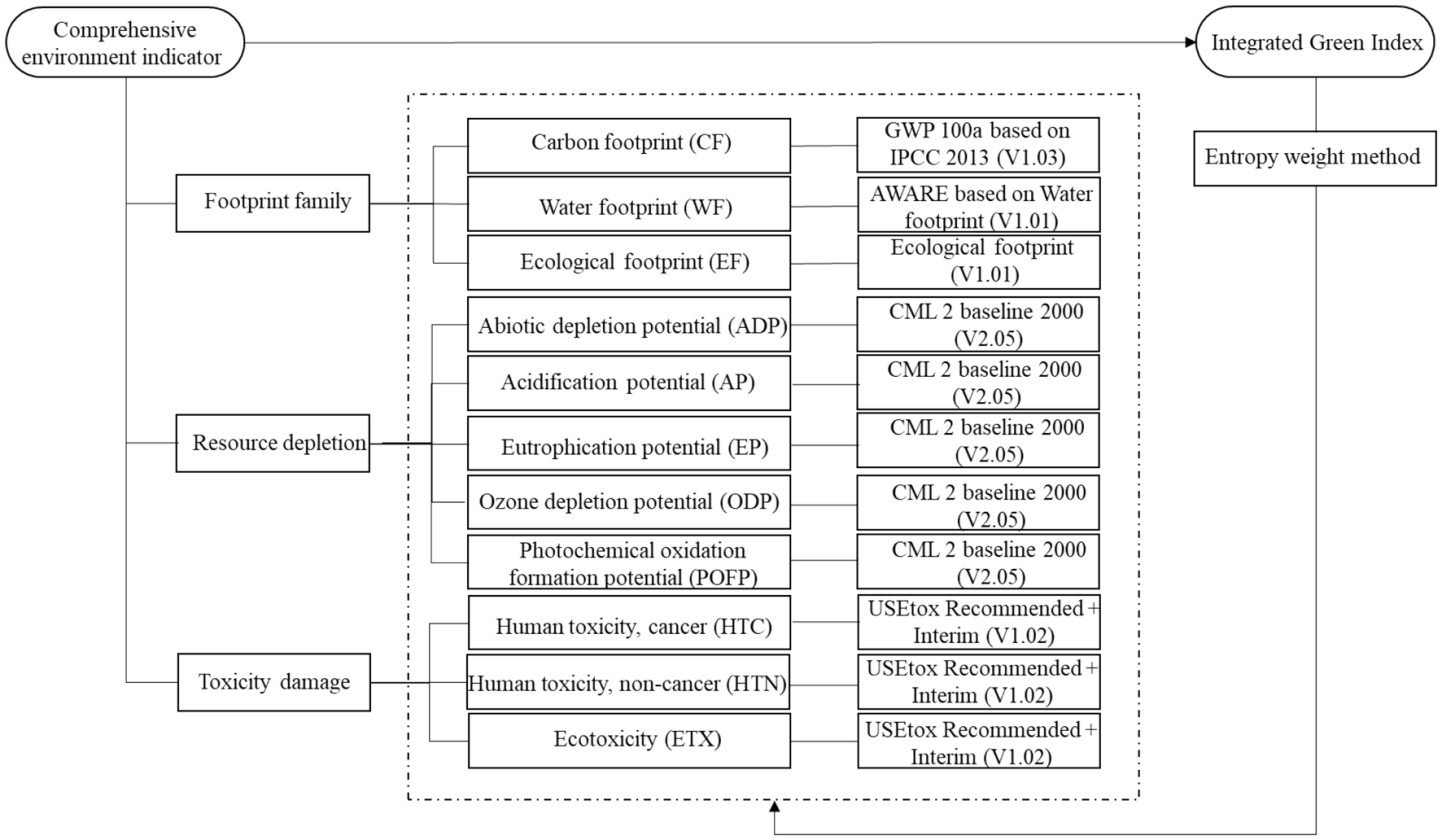
Comprehensive environmental assessment index.

In this study, by referring to domestic and foreign literature, 11 groups of representative three-level indicators were selected and divided into three groups of second-level comprehensive indicators: resource depletion, footprint family and toxic damage. The comprehensive environmental assessment index is shown in Fig. 3.

### Calculation of index weight

To evaluate the environmental characteristic of the battery pack as a whole, a comprehensive index, namely, the environmental characteristic index, was constructed on the basis of the second-level indicators, such as footprint family, resource depletion and toxic damage.

In the multi-index evaluation system, it is often inconvenient to compare and analyze the indexes because of the different units, dimensions and orders of magnitude of each index. Unified data processing can prevent different dimensions of the main indicators from affecting the evaluation results. As seen from the indicators of the comprehensive environmental evaluation system constructed, the indicators in the system are all reverse indicators, and the positive standardized formula is:

4 $${Z}_{ij}=\frac{\underset{1\ll i\ll n}{\mathrm{max}}{X}_{ij}-{X}_{ij}}{\underset{1\ll i\ll n}{\mathrm{max}}{X}_{ij}-\underset{1\ll i\ll n}{\mathrm{min}}{X}_{ij}};$$

In the formula, ${X}_{ij}$ represents the original data of the *j*th third-level index of the *i*th battery. *i* stands for different types of power packs (*i* = 1,2…11). *j* is the category of index data (*j* = 1, 2 … 11). ${Z}_{ij}$ is the standardized value of the *j*th index of the *i*th type battery. Among them, the value of ${Z}_{ij}$ ranges from 0 to 1. The larger the value is, the better the data of this indicator will be.

The entropy weight method is an objective weight method. In the specific process of use, the entropy weight of each index is calculated by using information entropy according to the degree of data dispersion of each index, and then the entropy weight is modified according to each index to obtain a relatively objective weight of the index. Entropy is used to measure the disorder degree of the system, as well as the effective information carried by the data, to determine the weight value of the index. If the information entropy of the index is smaller, it means that the variation degree of the index value is larger, and the information provided by the index is more, so it should play a greater role in the comprehensive evaluation, and the weight is higher. In this study, the introduction of weight did not change the basic research method, but sorted out the calculation results of LCA, to conduct an overall analysis of the battery pack environmental impact and make the results more accurate.

The information entropy of a set of data is:

5 $${S}_{j}=-\mathrm{ln}\left(\frac{1}{n}\right)\sum_{i=1}^{n}{P}_{ij}\mathrm{ln}{P}_{ij}$$

where $${P}_{ij}=\frac{{Z}_{ij}}{{\sum }_{i=1}^{n}{Z}_{ij}}$$, if $${P}_{ij}=0$$, $$\underset{{\mathrm{P}}_{\mathit{ij}}\to 0}{\mathrm{lim}}{P}_{ij}\mathrm{ln}{P}_{ij}=0$$

The corresponding weight of the indicator is:

6 $${y}_{j}=\frac{1-{S}_{j}}{m-{\sum }_{j=1}^{m}{S}_{j}}$$

where ${S}_{j}$ is the information entropy of a set of data and ${y}_{j}$ is the corresponding weight of the indicator.

**Figure 4:**
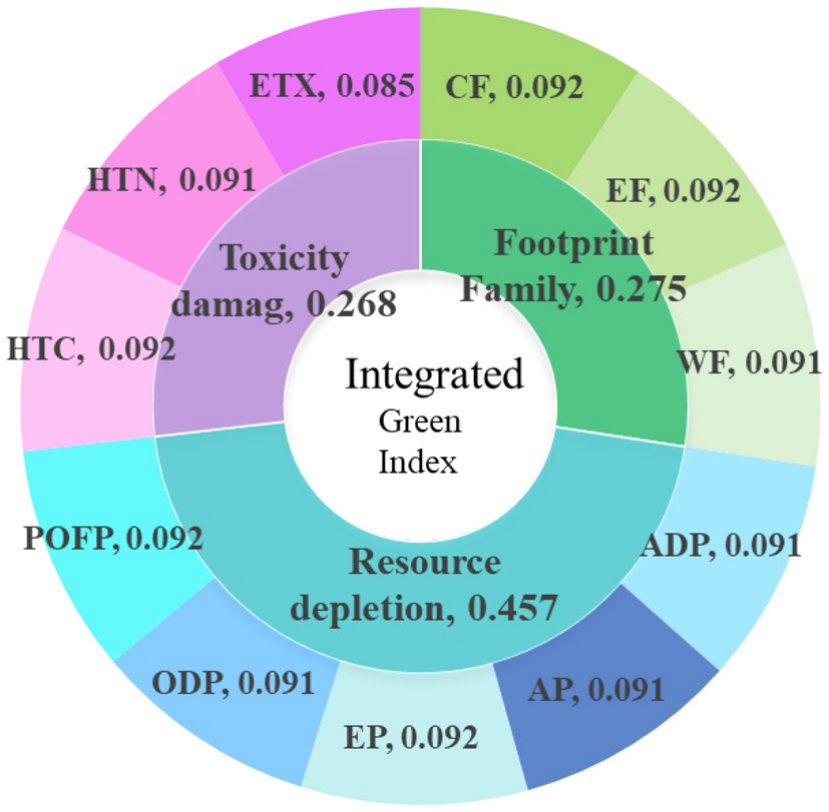
Indicator combinations and their weight values of global regional environmental characteristics indicators.

The entropy weight method is used to calculate the weight of each environmental index. Figure 4 shows the indicator combinations and their weight values of global regional environmental characteristic indicators.

Among the 11 third-level indicators, the weight value of the carbon footprint is the largest and that of POFP is the smallest, indicating that carbon footprint is one of the important reference indexes of environmental performance in the environmental impact assessment of battery packs.

The 11 impact indicators are the reflection of the battery emission potential in their respective fields. The environmental characteristic index reflects the comprehensive environmental impact of the battery pack in the use stage, that is, the cleanliness degree of the 11 impact indicators on the overall environmental condition. The higher the environmental characteristic index, the smaller the negative impact of the battery pack on the natural environment, that is, the cleaner the driving process. The calculation method of the environmental characteristic index is as follows:

7 $${E}_{i}=\sum_{j=1}^{m}{y}_{j}{Z}_{ij}$$

where ${E}_{i}$ is the environmental characteristic index of the *i*th battery pack.

## Discussion and results

**Figure 5:**
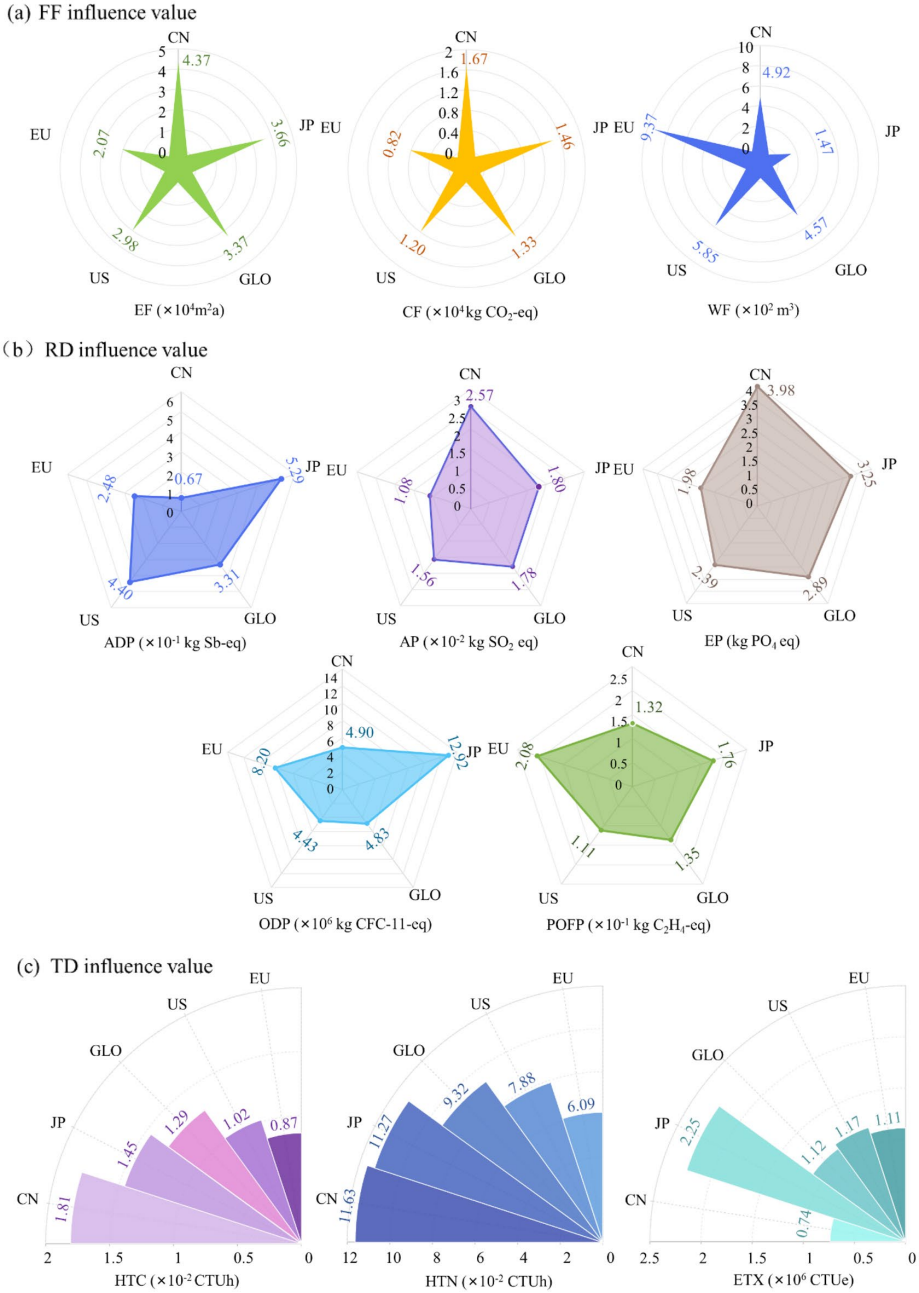
Footprint family, resource depletion, toxic damage influence value of mini vehicle battery pack in the use stage.

During the use stage of mini cars, the potential values of footprint family, resource depletion and toxic damage of all battery packs in the same area did not change significantly in the use stage. Therefore, the average value of each indicator of 11 kinds of batteries was taken for interregional comparison. In the use stage of the basic scenario, the environmental impact values of the footprint family, resource depletion, toxic damage generated by the battery pack of the mini model in the five regions are shown in Fig. 5.

### Footprint family influence at the battery pack use stage

As shown in Fig. 5a, EVs could indirectly cause different degrees of environmental impact due to the discrepancy in driving areas. The global average environmental impact value could be used as a reference standard to evaluate the environmental performance of vehicles in the use stage between regions. The Mini models in China and Japan could produce high carbon footprint (CF) and ecological footprint (EF) influence values, which were higher than the global standard. In contrast, the impact values of CF and EF generated in the European region were the lowest, which indirectly indicated that the use of EVs in China and Japan was not environmentally friendly. The reason was mainly due to the different regional power structures. For example, the coal power generation ratio in China was the highest among the five regions, at 66.54%, and the amount of greenhouse gases produced was higher than the global average. The regional patterns of EF and CF of the battery pack were similar. Therefore, China and Japan should optimize the power structure to further reduce greenhouse gas emissions.

In terms of the water footprint (WF), the only country with a lower global impact was Japan, while other countries or regions were above average in terms of water consumption, especially Europe and the US. This was mainly because nuclear power accounts for approximately one-fifth of the electricity mix in Europe and the US, compared with 10.15% globally. It could be speculated that the coal generation in the power structure could exert an influence on the CF and EF of the battery pack in the use stage. The nuclear structure could affect the EF of the battery pack during usage.

### The impacts of resource depletion during battery pack usage

As seen in Fig. 5b, the influence values of resource depletion indexes varied among different regions, and there was no consistency between small indexes. The battery pack of mini models running in China had the lowest value of abiotic depletion potential (ADP), while the battery pack of Japan had the highest value, indicating that China had better environmental performance in terms of ADP. In Japan and the US, the proportion of natural gas power generation was between 35 and 37%, higher than that in the other three regions, while it was only 3.28% in China. The proportion of natural gas power generation was similar to the influence value of ADP generated by EVs in the five regions. Therefore, it was speculated that natural gas generation in the power structure could affect the ADP of the battery pack during the running phase.

In different regions, the values of acidification potential and eutrophication potential generated by EV battery packs were consistent in regularity. The acidification potential (AP) and eutrophication potential (EP) values in China showed the highest state, while those of Europe were the lowest. Therefore, EVs in Europe and the US had better environmental performance in terms of acidification potential and eutrophication potential. For the power structure, China with a high coal power generation structure produces high acidification potential and eutrophication potential values, so it was speculated that coal power generation in the power structure could increase the acidification potential and eutrophication potential generated by the battery pack in the operation stage.

The ozone depletion potential (ODP) was close to the global average in China and the US, and the region with the highest value of ODP was Japan. The photochemical oxidant formation potential (POFP) value of EVs in Europe was the highest, which was quite low in the US. Affected by the law of environmental impact in each region, it was difficult to determine the real power generation structure leading to ozone depletion and photochemical oxidation, which might be the result of the synergistic effect of multiple power generation structures. Among the resource depletion indexes, the EV running in different regions had disparate environmental potential, and the values of acidification potential, eutrophication potential, ODP and POFP in the US were lower than the global average. In other words, the actual resource consumption of EVs in the transportation sector in the US was lower than the global average, which was most likely due to the relatively balanced power sources in the electric power structure of the US.

### Toxic damage effect of battery packs in the use stage

In the running stage of the basic scenario, the environmental impact value of toxic damage generated by the battery packs of the mini model in the five regions is shown in Fig. 5c. The variation trend of human toxicity cancer (HTC) and human toxicity non-cancer (HTN) in different regions was consistent, and the impact value of HTN in the corresponding assessment battery pack was 6–8 times higher than that of HTC on average. In the running stage, the EV battery packs of the mini model had the highest HTC and HTN impact value in China, but the lowest in Europe. For ecotoxicity (ETX), the conclusions were quite different. In the transportation sector, the actual ETX generated by EVs in Japan during operation could reach twice the global average value, belonging to the region with the highest ETX value, which was the lowest in China.

### Influence of the environmental characteristics of the battery pack in the use stage

**Figure 6:**
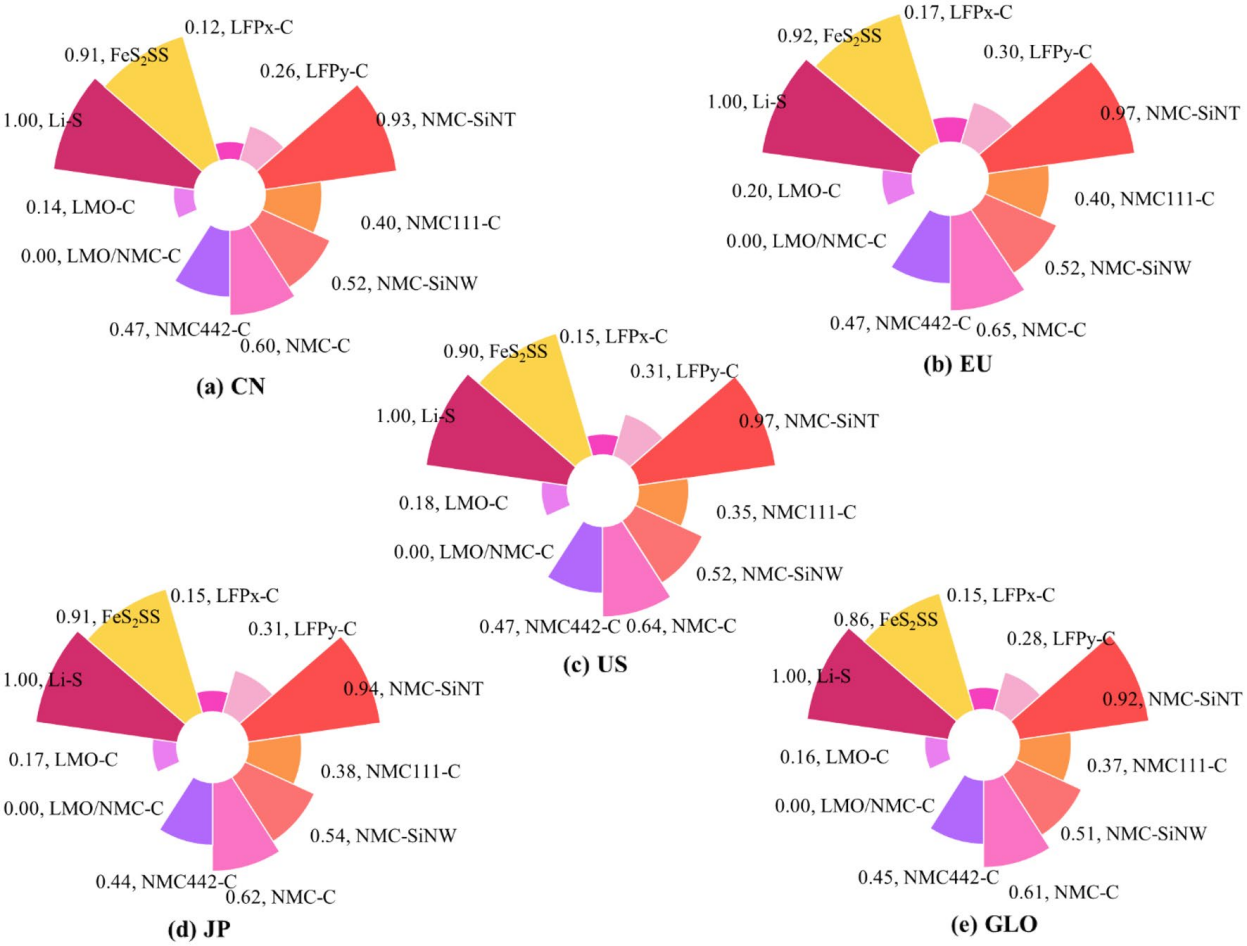
Environmental characteristic index of EVs with different battery packs in different areas.

According to the indirect environmental influence of the electric power structure, the environmental characteristic index could be used to analyze the environmental protection degree of battery packs in the vehicle running stage. The results showed that there was little difference in the environmental characteristic index of different areas, and the distribution law was roughly the same when different battery packs are assembled. The environmental characteristic index of EVs with different battery packs in different areas is shown in Fig. 6.

The environmental characteristic index is a positive index; the greater the value is, the better its environmental performance. Li–S battery pack was the cleanest, while LMO/NMC-C had the largest environmental load. The more electric energy consumed by the battery pack in the EVs, the greater the environmental impact caused by the existence of nonclean energy structure in the electric power composition, so the lower the environmental characteristics. In general, the battery pack's environmental characteristic index was sorted from large to small: Li–S, NMC-SiNT, FeS_2_SS, NMC-C, NMC-SiNW, NMC_442_-C, NMC_111_-C, LFP_y_-C, LFP_x_-C, LMO-C, LMO/NMC-C.

From the point of view of battery composition, the two LMB types of batteries have the highest environmental characteristics index (At the top of the list are Li–S batteries, with FeS_2_SS coming in third.), that is, it is the most clean and green during the use stage. This is mainly because such batteries have a higher energy density and a smaller weight for the same capacity, allowing them to consume less power on the road. In addition, NMC-SiNT has a higher mass energy density than FeS_2_SS. It can also be seen from Fig. 6 that the comprehensive environmental characteristic index of NMC-SiNT is greater than FeS_2_SS. In general, the mass energy density of NMC batteries is higher than that of LFP batteries, which is also the case in this paper. Therefore, the environmental characteristic indexes of LFP_y_-C and LFP_x_-C batteries are smaller than that of NMC batteries. Then, the comparative analysis of NMC battery shows that from the perspective of positive electrode, the molar ratio of nickel, manganese and cobalt components of NMC_442_-C and NMC_111_-C are different. Among them, NMC_442-_C has a higher nickel content than NMC111-C, resulting in a higher energy density and cleaner environmental impact. From the perspective of anode, the battery components of NMC-SiNT, NMC-C, NMC-SiNW three kinds batteries are different. NMC-SiNT uses silicon nanotubes as the negative electrode, NMC-C uses carbon as the negative electrode, and NMC-SiNW usessilicon nanowire as the negative electrode of the battery, which makes three batteries have different environmental characteristics. Finally, LMO batteries have the lowest environmental characteristic index,especially LMO/NMC-C, that is, they have the lowest environmental protection value.

### Development trend of the power structure in China

**Figure 7:**
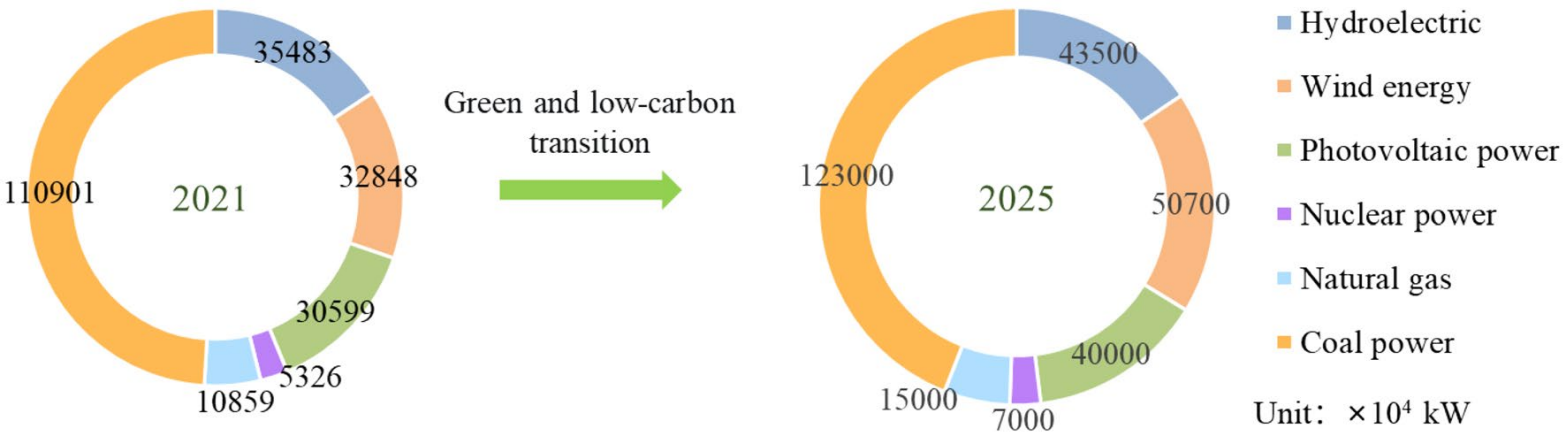
Installed power structure in 2021 and power trend in 2025.

In the past year, the global energy transition and energy shortage had been accompanied by each other. Countries around the world had tried their best to coordinate green and low-carbon energy development and energy supply. According to the Clean Energy Administration, the global demand for fossil fuels will peak within a few years, and the current energy crisis could be a turning point for clean energy development, accelerating the clean energy transition. The new trend of electric power development in China is shown in Fig. 7. The share of coal power installed in China is expected to drop from 49 percent in 2021 to 31 percent in 2025, which also follows a green and low-carbon transition. Therefore, clean transformation is the inevitable direction and the international trend of future energy development, and it is also the only way for China to change its high-carbon power supply structure.

Before 2020, power generation in China was dominated by coal which resulted in excess capacity for coal power generation and serious pollution. From 2021–2030, coal power will still play a basic role in coal conversion, electric heating supply and so on. Between 2031 and 2050, coal power will be simply an "adjustive power supply". With the goal of reaching the “carbon peak by 2030 and carbon neutral by 2060”, China will accelerate its clean transition and environmental development. Clean renewable energy will gradually become the majority of electricity supply. In other words, before 2030, China still relies heavily on coal power and is likely to maintain significant emissions from carbon footprint, ecological footprint, acidification potential, eutrophication potential, HTC and HTN, with improvements only in the clean-energy provinces. After 2030, the decreasing proportion of coal power will decrease the values of carbon footprint, ecological footprint, acidification potential and eutrophication potential. At the same time, clean and renewable energy becoming mainstay may greatly reduce HTN and HTC in China and enable EVs to drive cleanliness.

## Conclusion

In this paper, the effects of 11 groups of LIBs installed in mini EVs on 11 environmental indicators in 5 regions are discussed in detail. The results show that:

Battery packs operating in China will produce higher environmental potential value of CF, EF, AP, EP, HTC and HTN. Battery packs operating in Europe will produce higher WF and POFP, while operating in Japan will produce higher environmental impact value of ADP, ODP and ETX. The use of EVs has positive and significant environmental implications for above countries and regions in different aspects. EVs in Europe can best reduce of the value of CF, EF, HTC and HTN. EVs in Japan will lower the value of WF best. While in China, EVs best bring the reduction of the ADP and ETX. In order to reduce the ODP and POFP produced during operation, EVs are the best in the US.

From the environmental impact values of each region, it can be found that there is a correlation between the composition of power structure and environmental indicators. The coal power generation in the power structure can affect the CF, EF, AP and EP produced by the battery packs in the running stage. Nuclear power generation can affect the WF of the battery packs during operation. Natural gas power generation in the power structure can affect the ADP of the battery packs during operation. However, the green property index is a comprehensive index, which should not only consider the influence of the main power sources in the region, but also cannot ignore the role of other power generation modes. During the running phase, the battery pack with the highest environmental characteristic index is Li–S, while LMO/NMC-C has the lowest green characteristic index. This result occurs that the mass energy density is the key. With the same battery capacity and higher mass energy density, the weight of the battery pack is smaller, and the less electric energy needs to be consumed to carry the battery during the use stage, the more green the battery will be.

As can be seen from the research results, coal power in China currently has significant carbon footprint, ecological footprint, acidification potential and eutrophication potential, and its power structure is not conducive to the sustainable development of BEV. It is worth mentioning that China is striving to achieve a clean transition in power generation. In the future, it is hoped to establish a relationship model between power structure and impact index to simulate the impact of various changes of power structure on China's environment and find the optimal combination suitable for China's national conditions, so that BEV can drive green in China.

## Data Availability

The original contributions presented in the study are included in the article, further inquiries can be directed to the corresponding author.
